# Structural brain abnormalities in children and young adults with severe chronic kidney disease

**DOI:** 10.1007/s00467-021-05276-5

**Published:** 2021-11-20

**Authors:** Sophie Lijdsman, Marsh Königs, Marit S. van Sandwijk, Antonia H. Bouts, Koen van Hoeck, Huib de Jong, Marc Engelen, Jaap Oosterlaan, Frederike J. Bemelman, Kim J. Oostrom, Jaap W. Groothoff

**Affiliations:** 1 Department of Child and Adolescent Psychiatry & Psychosocial Care, Amsterdam Reproduction & Development, Emma Children’s Hospital, Amsterdam University Medical Centers (Amsterdam UMC), University of Amsterdam, G8-136, PO Box 22660, 1100 DD Amsterdam, Netherlands; 2 Emma Neuroscience Group, Department of Pediatrics, Amsterdam Reproduction & Development, Emma Children’s Hospital, Amsterdam UMC, University of Amsterdam, Amsterdam, Netherlands; 3 Department of Nephrology, Amsterdam Infection & Immunity, Amsterdam UMC, University of Amsterdam, Amsterdam, Netherlands; 4 Dianet Dialysis Centre, Amsterdam, Netherlands; 5 Department of Pediatric Nephrology, Amsterdam Reproduction & Development, Emma Children’s Hospital, Amsterdam UMC, University of Amsterdam, Amsterdam, Netherlands; 6 Department of Pediatrics, University Hospital Antwerp, Edegem, Belgium; 7 Department of Pediatrics, Sophia Children’s Hospital, Erasmus MC, Rotterdam, Netherlands; 8 Department of Pediatric Neurology, Amsterdam Reproduction & Development, Emma Children’s Hospital, Amsterdam UMC, University of Amsterdam, Amsterdam, Netherlands

**Keywords:** Chronic kidney disease, Magnetic resonance imaging, Adolescents, Kidney failure, Brain structure, Kidney replacement therapy

## Abstract

**Background:**

The pathophysiology of neurological dysfunction in severe chronic kidney disease (CKD) in children and young adults is largely unknown. We aimed to investigate brain volumes and white matter integrity in this population and explore brain structure under different treatment modalities.

**Methods:**

This cross-sectional study includes 24 patients with severe CKD (eGFR < 30) aged 8–30 years (median = 18.5, range = 9.1–30.5) on different therapy modalities (pre-dialysis, *n* = 7; dialysis, *n* = 7; transplanted, *n* = 10) and 21 healthy controls matched for age, sex, and parental educational level. Neuroimaging targeted brain volume using volumetric analysis on T1 scans and white matter integrity with tract-based spatial statistics and voxel-wise regression on diffusion tensor imaging (DTI) data.

**Results:**

CKD patients had lower white matter integrity in a widespread cluster of primarily distal white matter tracts compared to healthy controls. Furthermore, CKD patients had smaller volume of the nucleus accumbens relative to healthy controls, while no evidence was found for abnormal volumes of gray and white matter or other subcortical structures. Longer time since successful transplantation was related to lower white matter integrity. Exploratory analyses comparing treatment subgroups suggest lower white matter integrity and smaller volume of the nucleus accumbens in dialysis and transplanted patients relative to healthy controls.

**Conclusions:**

Young CKD patients seem at risk for widespread disruption of white matter integrity and to some extent smaller subcortical volume (i.e., nucleus accumbens). Especially patients on dialysis therapy and patients who received a kidney transplant may be at risk for disruption of white matter integrity and smaller volume of the nucleus accumbens.

**Graphical abstract:**

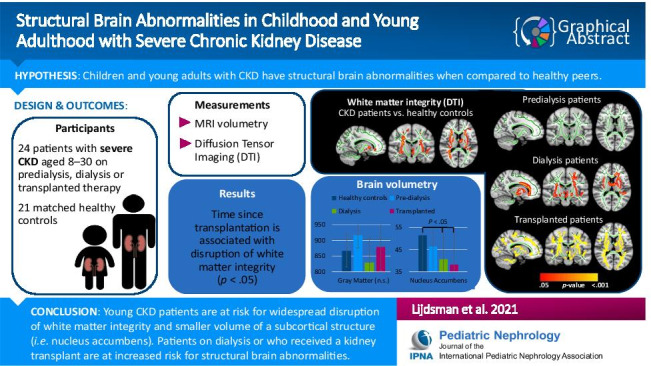

**Supplementary Information:**

The online version contains supplementary material available at 10.1007/s00467-021-05276-5.

## Introduction

Severe chronic kidney disease (CKD) in children and young adults impairs neurocognitive and psychosocial development, characterized by behavioral, social, learning, and vocational problems [1–8]. CKD-induced structural brain abnormalities have been suggested to underlie these disabling difficulties in daily life [5, 9]. To date, it is not entirely clear which factors affect the developing brain in patients with CKD. High levels of uremic neurotoxins, osmotic shifts induced by hemodialysis, and cerebral perfusion impairment imposed by hemodialysis and/or immunosuppressive medication in transplanted patients are all considered among the potential pathophysiological mechanisms [9–14].

Studies on the pathophysiology of neurological dysfunction in adults with severe CKD reported smaller brain volumes on magnetic resonance imaging (MRI) scans, indicative of brain atrophy [15–22]. Diffusion tensor imaging (DTI) is an advanced MRI method that is particularly sensitive to the microstructure of white matter tracts [23]. Studies using DTI showed disrupted white matter integrity in adult patients with severe CKD [16, 20, 21, 24–26]. Comparisons of treatment subgroups in adults further indicate that dialysis patients are particularly at risk of brain atrophy and reduced white matter integrity, while the anomalies in transplanted patients are less pronounced [15–22, 27, 28]. Pre-dialysis patients appear to be at the lowest risk of brain abnormalities [28]. Longitudinal studies in transplanted adult patients yield inconsistent findings, with some studies showing improvement and others showing further deterioration of MRI parameters after transplantation [9, 27, 29, 30]. Higher uremic toxin levels and longer dialysis duration were related to more severe brain atrophy and disruption of white matter integrity in adults [18, 21, 22, 24, 26, 31]. Taken together, previous studies indicate that CKD and kidney replacement therapy are related to structural abnormalities in the adult brain. As rapid brain development takes place well into early adulthood [32], children and young adults may be particularly sensitive to the detrimental effects of CKD on the developing brain. Indeed, the limited available studies in young CKD patients indicate that CKD may affect normal brain development [9, 33–35]. The exact mechanisms of CKD that impact on the developing brain remain unclear however, and studies comparing patients on conservative therapy, dialysis, or living with a functional kidney graft are lacking in particular.

The current MRI study aims to explore the impact of (1) CKD on brain structure (as quantified by brain volumetry and tract-based spatial statistics on DTI as a measure of white matter microstructural integrity) and (2) different treatment modalities on brain structure. Based on the available literature in adults, it was expected that children and young adults with severe CKD had smaller brain volumes and lower white matter integrity compared to matched controls, where patients on dialysis therapy would be particularly at risk.

## Methods

### Participants

We included 24 patients with severe CKD aged 8.0–30.9 years and 21 healthy controls matched for age, sex, and parental educational level. CKD patients were recruited from the Amsterdam University Medical Centers (*n* = 21); Erasmus Medical Centre, Netherlands (*n* = 1); and the University Hospital Antwerp, Belgium (*n* = 2). Inclusion criteria for the CKD group were (1) CKD stages 4–5 on conservative therapy, on peritoneal or hemodialysis or patients having received a kidney transplant at least two years prior to enrollment (to ensure stable kidney function), and (2) aged between 8 years (in line with national ethical guidelines) and 30 years. Healthy controls were recruited through participating patients (friends or acquaintances, not siblings) or through local schools and sport clubs. For both patients and controls, exclusion criteria were (1) previously established severe intellectual impairment with overt learning disability; (2) insufficient mastery of the Dutch language; (3) primary sensory disorder (hearing or vision impairments); (4) established skull or brain abnormalities not related to CKD; or (5) co-existing disease with primary or secondary central nervous system involvement interfering with the impact of CKD.

### Treatment subgroups

Three treatment subgroups of CKD patients were distinguished: (1) a pre-dialysis group (*n* = 7) with current estimated glomerular filtration rate (eGFR) < 30 ml/min/1.73 m^2^ on conservative treatment at time of assessment; (2) a group on chronic hemodialysis or peritoneal dialysis (total *n* = 7, hemodialysis *n* = 2, peritoneal dialysis *n* = 5); and (3) a transplanted group (*n* = 10) of patients with a functioning kidney graft for at least 2 years and eGFR > 30 ml/min/1.73 m^2^ [36]. CKD patients who previously underwent kidney transplantation, but had an eGFR < 30 at time of assessment, were allocated to either the pre-dialysis or dialysis group according to their current treatment mode.

### Measurements

#### Socio-demographic and CKD clinical parameters

Socio-demographic parameters (i.e., age, sex, parental educational level) were collected via an online portal [37], using a self-developed widely used custom-made inventory. Parental educational level was divided into three categories: (1) low education (primary education, lower vocational education, lower and middle general secondary education); (2) middle education (middle vocational education, higher secondary education, pre-university education); and (3) high education (higher vocational education, university) [38]. The following clinical parameters were extracted from each patient’s medical file: age at severe CKD diagnosis (when eGFR first dropped below 30 ml/min/1.73 m^2^), primary disease, history of relevant comorbidities (extreme prematurity [< 32 weeks of gestational age], malignant hypertension [extremely high blood pressure resulting in organ damage], and convulsions), current eGFR, creatinine and urea blood levels obtained closest to the date of study participation (range of lab measurements: − 51 days to + 4 days relative to MRI scan), duration of severe CKD (ratio of the time frame between the moment that eGFR dropped below 30 ml/min/1.73 m^2^ and the time of assessment [months] to calendar age [months], expressed as % of life), type of dialysis received during lifetime (hemodialysis, peritoneal dialysis, or both), dialysis duration (ratio of dialysis duration [months] to calendar age [months], i.e., % of life), type of transplantation (pre-emptive or non-pre-emptive) and time since successful transplantation (ratio of period since transplantation during which eGFR > 30 ml/min/1.73 m^2^ [months] to calendar age [months], i.e., % of life). eGFR was calculated using Schwarz formula for patients aged < 18 years [39] and the abbreviated Modification of Diet in Renal Disease formula was used for patients aged > 18 [40]. Due to large fluctuations in eGFR prior to and after dialysis, eGFR of patients receiving dialysis was conservatively set at 10 [41].

### MRI acquisition and pre-processing

MRI scans were acquired on a 3.0 T Philips Achieva scanner using a 32-channel head coil. T1-weighted and spin echo diffusion-weighted images using 128 diffusion gradient directions were acquired. Pre-processing of T1 data produced normalized brain volumes of gray matter, white matter, and bilateral subcortical structures (i.e., thalamus, caudate nucleus, putamen, pallidum, hippocampus, amygdala, and nucleus accumbens). Pre-processing of diffusion-weighted images produced maps for fractional anisotropy (FA) and mean diffusivity (MD) as the primary measures of white matter integrity (higher FA and lower MD values are consistent with higher integrity [23]). Secondary measures were axial diffusivity (AD) and radial diffusivity (RD), for which a pattern of lower AD and higher RD is consistent with axonal degeneration and/or demyelination [23]. Further details on the MRI acquisition and preprocessing are provided in Supplement 1.

### Procedure

The study protocol was approved by the Medical Ethics Committee of the Amsterdam UMC (NL61708.018.17), and all procedures were performed according to the Declaration of Helsinki. Eligible patients were first approached by their treating physician. Eligible controls were approached by the researcher and received a flyer. Those who responded positively were again contacted, either in person or by phone, for additional information and received a comprehensive information letter. After 2 weeks, potential candidates were re-contacted by telephone to answer remaining questions. After obtaining verbal consent to participate, written informed consent was obtained from legal guardians (for children aged < 16 years old) and/or children and young adults aged ≥ 12 years.

One week prior to the MRI scan, participants and/or parents of participants < 18 years old completed the online questionnaires. The 30-min MRI scan took place at the Amsterdam Medical Centre. If children expressed that they wanted to become familiar with scanning procedures, a simulation scanner was available and a mock procedure was performed before actual scanning.

### Statistical analyses

#### Socio-demographic and clinical characteristics

Statistical analyses were performed using SPSS 26.0 (IBM Corp., 2019). Independent and dependent variables were tested for normality and screened for outliers (± 3 interquartile ranges below/above the lower or upper quartile), which were rescaled using winsorizing (Field, 2009). All groups (CKD group, healthy control group, and treatment subgroups) were compared with each other by age, sex, and parental educational level and clinical parameters using analysis of variance (ANOVA).

#### Analysis of possible confounders

Matching between the total CKD and the healthy control group by age, sex, and parental educational level during recruitment controlled for possible confounding effects of socio-demographic factors in these analyses [32, 42]. With regard to treatment subgroup comparisons and analyses of clinical parameters, we explored the association between socio-demographic parameters and all outcome measures using correlation analyses (age), *t* tests (sex), and ANOVAs (parental educational level). Socio-demographic parameters that showed a significant relationship with a particular outcome measure were added as covariates to treatment subgroup comparisons and analyses of clinical parameters on these specific outcomes.

#### Brain volumes in CKD groups and healthy control group

Regarding brain volume, group differences (CKD group, healthy control group) were assessed and treatment subgroup differences (pre-dialysis group, dialysis group, transplanted group, healthy control group) were explored using ANOVA. Main effects of treatment subgroup were followed by planned contrasts comparing each treatment subgroup to the healthy control group. Group differences in brain volumes between non-pre-emptively and pre-emptively transplanted patients were explored using *t* tests. The relationship between clinical parameters (age at severe CKD diagnosis, current eGFR, severe CKD duration, dialysis duration, and time since successful transplantation) was investigated using multivariate linear regressions with backward elimination (criterion for removal: *p* > 0.10) on brain volumes for which a significant effect of treatment subgroup was found, in order to reduce amount of comparisons.

#### White matter integrity in CKD groups and healthy control group

Statistical analyses of DTI maps were performed using *randomize* [43]. Group differences and treatment subgroup differences were evaluated using voxel-wise comparisons of the skeletonized FA and MD maps. Group differences between the non-pre-emptive and pre-emptive group were also explored. The relation between clinical parameters and white matter integrity was investigated using voxel-wise regression on FA and MD maps. Only in case a significant cluster was identified for FA or MD maps, the origin of the impact of CKD was further investigated by comparing the mean AD and RD extracted from the cluster affected by CKD.

Finally, to identify the white matter tracts contributing to the neuropathology of CKD, masks of (bilateral) white matter tracts were created for the following tracts: genu, body and splenium of corpus callosum (CC), corticospinal tract (CST), anterior thalamic radiation (ATR), superior and longitudinal fasciculus (SLF and ILF), inferior frontal occipital fasciculus (IFOF), forceps major and minor (FMa and FMi), cingulate and hippocampal parts of the cingulum bundle (CB), and uncinate fasciculus (UF). Overlap between each white matter tract and the cluster affected by CKD was used to assess (1) which white matter tracts contributed to the impact of CKD (i.e., percentage overlap of each white matter tract with the total cluster affected by CKD) and (2) to what extent each white matter tract was affected by CKD (i.e., percentage overlap of affected cluster with each complete white matter tract).

All statistical testing was two-sided and alpha was set at 0.05. Cohen’s *d* effect sizes are reported where appropriate and were interpreted as small (*d* < 0.5), medium (0.5 ≥ *d* < 0.8), or large (*d* ≥ *0.80)* [44].

## Results

### Socio-demographic and clinical characteristics

Socio-demographic and clinical characteristics of the sample are shown in Table 1. Comparisons between the healthy control group, the CKD group, and the treatment subgroups revealed no significant differences on any of the socio-demographic parameters. Regarding clinical parameters, age at severe CKD diagnosis was significantly higher in the dialysis group than in the transplanted group (*p* = 0.015,* d* = 1.41). As expected, treatment subgroups differed in terms of eGFR and blood urea level, where the transplanted group had higher eGFR and lower blood urea levels than both the pre-dialysis and dialysis group (*p*s < 0.001, *d*s > 2.16), while the pre-dialysis group had lower blood urea levels than the dialysis group (*p* = 0.029, *d* = 1.20). As expected, time since successful transplantation was longer in the transplanted group than in the pre-dialysis and dialysis group (*p* < 0.001, *d* = 2.07 and *p* < 0.001, *d* = 2.09, respectively). Other comparisons on clinical parameters did not reveal significant differences.

**Table 1:** Demographic and clinical parameters in the CKD, pre-dialysis, dialysis, transplanted, and healthy control group

	Group		Contrasts		Treatment subgroups			Statistics	
	CKD	Healthy controls	*p*	*d*	Pre-dialysis	Dialysis	Transplanted	*p*	Contrasts
*n*	24	21			7	7	10		
Age	18.5 (9.1–30.5)	19.8 (9.0–29.2)	0.933	0.03	15.5 (9.1–26.6)	21.3 (9.6–27.1)	17.8 (9.1–30.5)	0.310	
Male	*n* = 16 (66.7%)	*n* = 13 (61.9%)	0.746	–0.10	*n* = 6 (85.7%)	*n* = 3 (42.9%)	*n* = 7 (70.0%)	0.249	
Educational level of parents^a^	2.0 (1.0–3.0)	2.0 (1.0–3.0)	0.890	0.05	2.0 (2.0–3.0)	2.0 (1.0–3.0)	2.0 (1.0–3.0)	0.661	
Age at severe CKD diagnosis (years)	14.0 (0.0–24.7)				13.8 (1.9–24.7)	20.2 (9.3–24.0)	8.1 (0.0–22.8)	**0.049**	D > Tx
Primary disease (*n*)									
• CAKUT^1^ • Renovascular^2^ • Cortical necrosis^3^ • Acquired glomerulopathy^4^ • Inherited nephropathy^5^ • Other and unknown cause^6^	7 2 3 2 7 3				3 - - 1 2 1	- - 2 1 3 1	4 2 1 - 2 1		
History of relevant comorbidities									
• Extreme prematurity (< 32 weeks of GA)	1				-	-	1		
• Malignant hypertension • Convulsions/history of epilepsy	5 2				- -	2 1	3 1		
eGFR (ml/min/1.73 m^2^)	26.6 (10.0–90.0)				24.8 (11.3–29.0)	10.0 (10.0–10.0)	52.9 (31.0–90.0)	** < 0.001**	Tx > PD & D
Urea (mmol/L)	14.9 (5.1–25.1)				16.8 (13.7–20.7)	20.7 (16.8–28.8)	7.6 (5.1–13.3)	** < 0.001**	D > PD > Tx
Duration severe CKD (% of life)	12% (0–81%)				11% (0–81%)	8% (3–21%)	20% (4–78%)	0.347	
Ever treated by dialysis (*n*) Hemodialysis Peritoneal dialysis Both	14 5 8 1				2 2 - -	7 2 5 -	5 1 3 1		
Duration dialysis (% of life)	1% (0–49%)				0% (0–6%)	4% (1–14%)	2% (0–49%)	0.371	
Ever treated by kidney transplantation (*n*)	13				2	1	10		
Pre-emptive Non-pre-emptive	6 7				1 1	- 1	5 5		
Time since kidney transplantation (% of life)	6% (0–61%)				0% (0–6%)	0% (0–10%)	23% (13–61%)	** < 0.001**	Tx > PD & D

Values are displayed as median (range), unless otherwise indicated. Abbreviations: CKD = chronic kidney disease; D = dialysis group; eGFR = estimated glomerular filtration rate; GA = gestational age; PD = pre-dialysis group; Tx = transplanted group. ^a^ 1.0 = low education, 2.0 = middle education, 3.0 = high education

Primary diseases: ^1^ urethral valves (*n* = 6), dysplasia (*n* = 1), ^2^ malignant hypertension (*n* = 2), ^3^ due to asphyxia (*n* = 1), due to septicemia (*n* = 2); ^4^ primary focal segmental glomerulosclerosis (FSGS) (*n* = 1), anti-neutrophil cytoplasmic autoantibodies (ANCA) vasculitis (*n* = 1); ^5^ branchiootorenal (BOR-) syndrome (*n* = 1), *NPHP1* mutation (*n* = 1), autosomal dominant polycystic kidney disease (ADPKD) (*n* = 1), Alport’s syndrome (*n* = 1), inherited FSGS due to *INF2* mutation (*n* = 2), *Pax-2* mutation (*n* = 1); ^6^ tubulointerstitial nephritis (*n* = 1), unknown cause (*n* = 2)

### Brain volumes in CKD groups and healthy control group

Table 2 provides the results of the volumetric analysis. As compared to the healthy control group, the CKD group had smaller volume of the nucleus accumbens (*p* = 0.005, *d* =  − 0.87). No further significant differences between the CKD group and healthy control group were found for volumes of the gray matter, white matter, or subcortical structures (*p*s > 0.072). Exploratory analyses comparing treatment subgroups showed a significant main effect of treatment type for volume of the nucleus accumbens (*p* = 0.022). No significant main effect of treatment type was found for volumes of gray and white matter or other subcortical structures (*p*s > 0.222). Subsequent analyses revealed that, as compared to the healthy control group, both the dialysis and transplanted groups had smaller nucleus accumbens volume (*p* = 0.037,* d* =  − 0.94; *p* = 0.005, *d* =  − 1.28, respectively). Group differences between the non-pre-emptively transplanted and pre-emptively transplanted group were not significant (*p*s > 0.183, *d*s < 0.79; Table 3).

**Table 2:** Brain volumes in the CKD, pre-dialysis, dialysis, transplanted, and healthy control group

n	Group		Contrasts		Treatment subgroups			Statistics^a^		
	CKD	Healthy control	*p*	*d*	Pre-dialysis	Dialysis	Transplanted	*p*	Contrasts	Covariates
	24	21			7	7	10			
*Brain volume (cm*^*3*^*)*										
Gray matter	875.8.8 (84.7)	866.9 (61.4)	0.690	0.12	916.5 (84.8)	829.4 (60.9)	879.9 (89.8)	0.323	-	Age: *p* < 0.000,
White matter	750.5 (35.9)	752.6 (32.5)	0.839	− 0.06	739.3 (24.5)	757.0 (16.7)	753.7 (50.6)	0.777	-	n.s.
*Subcortical volume (cm*^*3*^*)*										
Thalamus	574.4 (51.3)	546.2 (51.3)	0.072	0.55	576.1 (47.6)	557.2 (46.9)	585.4 (58.1)	0.222	-	n.s.
Caudate nucleus	300.9 (40.4)	312.3 (34.5)	0.316	− 0.30	293.2 (36.3)	310.5 (32.6)	299.6 (49.6)	0.633	-	n.s.
Putamen	431.2 (47.9)	429.5 (33.8)	0.893	0.04	428.4 (33.0)	409.0 (39.2)	448.6 (58.7)	0.289	-	n.s.
Pallidum	146.0 (15.8)	150.1 (15.4)	0.388	− 0.26	143.0 (9.8)	149.7 (18.4)	145.4 (18.1)	0.710	-	n.s.
Hippocampus	329.7 (46.6)	339.3 (31.7)	0.431	− 0.24	328.2 (39.7)	348.8 (65.0)	317.4 (35.2)	0.376	-	
Amygdala	78.5 (30.6)	86.7 (27.5)	0.350	− 0.28	73.4 (28.9)	71.4 (37.3)	87.0 (27.8)	0.513		n.s.
Nucleus accumbens	41.3 (13.3)	51.5 (9.5)	**0.005**	− 0.87	46.4 (11.3)	40.6 (16.9)	38.2 (12.1)	**0.022**	HC > D & Tx	n.s.
*DTI parameters*										
FA in CKD cluster	0.462 (.0014)	0.498 (0.020)	** < 0.001**	− 2.10	0.473 (0.014)	0.463 (0.013)	0.455 (0.013)	** < 0.001**	HC > D & Tx	Age: p = 0.020
MD in CKD cluster (10^–5^ mm^2^/s)	78.7 (1.9)	77.3 (1.9)	**0.018**	0.074	79.0 (1.7)	78.2 (1.7)	79.0 (2.2)	**0.034**	HC < Tx	Age: p < 0.001

Means and standard deviations are displayed. Abbreviations: CKD = chronic kidney disease; D = dialysis group; DTI = diffusion tensor imaging; FA = fractional anisotropy; HC = healthy controls; MD = mean diffusivity; PD = pre-dialysis group; Tx = transplanted group

^a^ *p* values and Cohen’s *d* effect sizes for treatment group comparisons using AN(C)OVA are provided and post hoc contrasts of ANOVA are used

**Table 3:** Brain volumes and DTI parameters in non-pre-emptive and pre-emptive transplantation groups

*n*	Groups		Contrasts	
	Non-pre-emptive	Pre-emptive	*p*	*d*
	7	6		
*Brain volume (cm*^*3*^*)*				
Gray matter	858.9 (93.6)	909.4 (102.3)	0.373	− 0.52
White matter	757.3 (23.7)	741.1 (44.7)	0.538	− 0.46
Thalamus	603.9 (46.9)	563.2 (56.6)	0.183	0.79
Caudate nucleus	300.3 (51.3)	300.4 (50.9)	0.996	0.00
Putamen	447.6 (42.9)	443.5 (68.0)	0.897	0.07
Pallidum	148.5 (13.6)	143.3 (19.8)	0.590	0.31
Hippocampus	331.9 (52.5)	329.2 (31.5)	0.915	0.06
Amygdala	76.1 (26.1)	89.1 (34.9)	0.456	− 0.43
Nucleus accumbens	37.9 (13.0)	44.4 (13.6)	0.396	− 0.49
*DTI parameters*				
FA in CKD cluster	0.458 (0.011)	0.453 (0.013)	0.495	0.42
MD in CKD cluster (10^–5^ mm^2^/s)	78.6 (1.7)	79.6 (2.2)	0.477	− 0.51

Means and standard deviations are displayed. Abbreviations: CKD = chronic kidney disease; DTI = diffusion tensor imaging; FA = fractional anisotropy; MD = mean diffusivity

Multiple regression analysis in the total CKD group did not reveal a significant association between clinical parameters and the subcortical structure with an observed effect of treatment subgroup (i.e., nucleus accumbens).

### White matter integrity in CKD groups and healthy control group

**Fig. 1:**
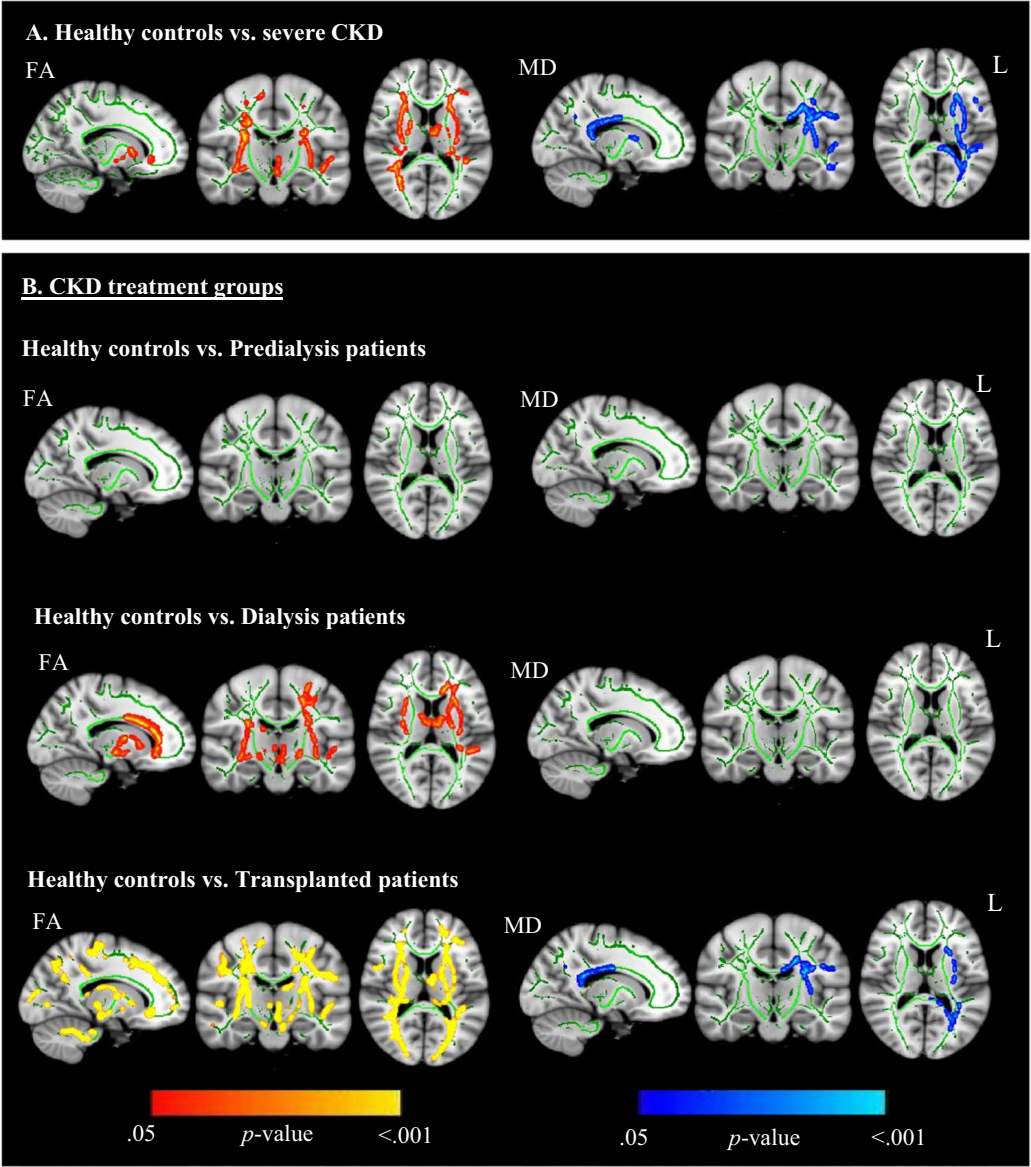
Illustration of voxel-wise TBSS comparisons of FA and MD maps, using threshold-free cluster enhancement correction, showing significantly lower white matter integrity in CKD patients (**A**) and dialysis and transplanted patients (**B**) compared to healthy controls. For treatment subgroup comparisons, age was included as covariate to control for confounding effects, as age varied among treatment subgroups and had a significant effect on FA and MD maps. Red–yellow clusters show reduced FA values in the CKD groups compared to the healthy controls. Blue–lightblue clusters show increased MD values in the CKD groups. More yellow and lighter blue areas indicate higher significant values. All images are illustrated at the same coordinates (*x* =  − 15, *y* =  − 16, z = 13) and show the whole brain skeleton (at FA > 0.3, in green), overlaid on standard MNI 152 1 mm T1 brain. Significant group differences are “thickened” towards the full width of the white matter tract to increase visualization. Abbreviations: CKD = chronic kidney disease; FA = fractional anisotropy; MD = mean diffusivity; L = left

Voxel-wise group comparisons revealed that the CKD group had lower FA and higher MD than the healthy control group in a large cluster of white matter tracts (*p* < 0.001, *d* = –2.10; *p* = 0.018, *d* = 0.74, respectively), as displayed in Fig. 1a. Subsequent analyses showed that within this affected cluster, the CKD group had higher RD (*p* < 0.001, *d* = 1.52) and lower AD (*p* = 0.002, *d* = –1.01) as compared to the healthy control group. When considering individual white matter tracts, the IFOF, ATR, and SLF had the highest contribution to the cluster affected by CKD and the CC, CB, FMa, and FMi had least or no contribution. The IFOF, UF, ATR, and CST were most extensively affected by CKD and the CC and CB were not or barely affected (Table 4).

**Table 4:** White matter tracts involved in the impact of severe CKD

White matter tract	Percentage of cluster	Percentage of tract affected
Inferior frontal occipital fasciculus (IFOF)	23.8	26.7
Anterior thalamic radiation (ATR)	12.6	19.1
Superior longitudinal fasciculus (SLF)	11.5	13.8
Cortical spinal tract (CST)	9.2	18.6
Uncinate fasciculus (UF)	6.4	24.5
Inferior longitudinal fasciculus (ILF)	4.4	9.2
Forceps minor (FMi)	1.3	3.1
Forceps major (FMa)	1.2	4.2
Splenium of corpus callosum	0.1	0.6
Body of corpus callosum	0.1	0.4
Hippocampal part of cingulum bundle (HCB)	0.0	0.0
Cingulate part of cingulum bundle (CCB)	0.0	0.0
Genu of corpus callosum	0.0	0.0

**Fig. 2:**
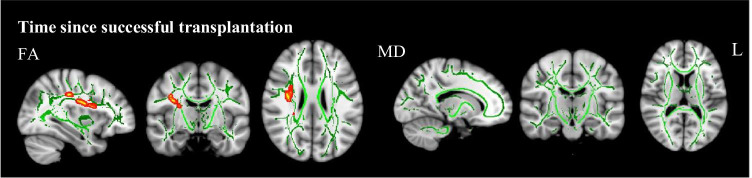
Illustration of voxel-wise TBSS comparisons of FA and MD maps, using threshold-free cluster enhancement correction, showing a significant negative correlation between time since succesful transplantation and FA values in CKD patients. Image is illustrated the following coordinates: (*x* = 34, *y* =  − 10, *z* = 26) and shows the whole brain skeleton (at FA > 0.3. in green), overlaid on standard MNI 152 1 mm T1 brain. Significant group differences are “thickened” towards the full width of the white matter tract to increase visualization. Abbreviations: FA = fractional anisotropy; MD = mean diffusivity; L = left

Exploratory voxel-wise group comparisons of the treatment subgroups on FA and MD maps showed no difference between the pre-dialysis group and healthy control group, while the dialysis group had lower FA in a large cluster of white matter tracts compared to the healthy control group (*p* < 0.001, *d* =  − 1.92) (Fig. 1b). The transplanted group had both lower FA and higher MD in a large cluster of white matter tracts (*p* < 0.001, *d* =  − 2.40; *p* = 0.034, *d* = 0.81, respectively). Follow-up analyses on AD and RD maps showed that within the cluster of affected white matter, both the dialysis and transplanted group had higher RD (*p* = 0.005, *d* = 1.30; *p* < 0.001 *d* = 1.68, respectively) and lower AD (*p* = 0.003, *d* = –1.52; *p* = 0.002, *d* = –1.27, respectively) compared to the healthy control group. No significant differences were found between the non-pre-emptive and pre-emptive transplantation group. Voxel-wise regression analysis revealed that longer time since successful transplantation was significantly related to lower FA (*β* =  − 0.518, *p* = 0.011, while no relations were found with MD, RD, and AD (Fig. 2). No other significant relations between clinical parameters and white matter integrity were revealed.

## Discussion

The results of this exploratory MRI study suggest that CKD patients aged 8 to 30 years are at risk for widespread abnormality of DTI parameters in the cerebral white matter. These findings are consistent with the hypothesis that CKD results in disruption of white matter integrity indicative of axonal damage and/or demyelination. White matter abnormalities may contribute to the presence of neurocognitive impairments that are considered prominent in the clinical features of CKD [2–7]. No evidence was found for smaller brain volume of the gray and white matter, although there were indications for smaller volume of a subcortical structure (i.e., nucleus accumbens). Our findings further suggest that longer time since successful transplantation may be related to more severe disruption of white matter integrity. Additional exploratory comparisons between treatment subgroups further suggest that dialysis and transplanted patients may both be vulnerable for disruption of white matter integrity and to some extent smaller subcortical volumes (i.e., in the nucleus accumbens), with no evident differences between the two groups. In contrast, no evidence was found for structural brain abnormalities in pre-dialysis patients with severe CKD.

Findings from DTI analyses suggest that our patients have widespread disruption of white matter integrity. The observed effect of severe CKD on white matter integrity is in line with previous studies showing a negative impact of CKD on the adult brain [16, 20–22, 24–26] and the only existing DTI study in young CKD patients that found relatively modest abnormality of white matter integrity [33]. Our study extends the existing literature by showing that the negative impact of CKD on white matter tracts in young patients is widespread, where the larger distal white matter tracts (i.e., IFOF, ATR, SLF, CST, and UF) seem more prominently involved than medial white matter tracts (i.e., CC, CB, FMA, and FMi). Additionally, findings from volumetric analysis further indicate that young patients with severe CKD may have smaller volume of the nucleus accumbens, while no evidence was found for smaller brain volume of gray and white matter or other subcortical structures. These findings contribute to already inconsistent literature on the impact of CKD on brain volumes of young patients [34, 35]. The nucleus accumbens is a subcortical structure involved in behavioral arousal and the regulation of slow-wave sleep [45]. This may suggest that abnormal development of this structure plays a role in the clinical presentation of CKD in young patients, as sleep is often disturbed in these patients [46]. The observed effect on the nucleus accumbens contrasts with the single previous study on subcortical volumes in young patients, reporting no effects of CKD on subcortical volumes [34]. This discrepancy may be explained by higher CKD severity in our study sample (eGFR < 30, while also including dialysis patients). However, our results are in line with a study in older dialysis patients, which also reported smaller volume of the nucleus accumbens [16]. Taken together, these findings may support the idea that subcortical structures may be more vulnerable in patients with more severe stages of CKD. To conclude, our combined results of DTI and volumetric analyses indicate that abnormal white matter integrity is prominently implicated in the effect of CKD on the young brain. In addition, there is some evidence indicating a negative effect on subcortical structures, more specifically the nucleus accumbens.

Exploratory analyses comparing treatment subgroups indicate that dialysis patients have structural brain abnormalities (i.e., disruption of white matter integrity and smaller volume of the nucleus accumbens) relative to unaffected peers, while no evidence was found for brain abnormalities in pre-dialysis patients. This is in line with previous studies in children and young adults with CKD on conservative therapy [33, 34] and adults on dialysis therapy [3, 9, 16, 25–27, 31]. No associations were found between dialysis duration and severity of the observed brain abnormalities. Although other studies in adults have reported associations between dialysis duration and the volume of gray and white matter [18, 27, 28, 31], the results from this study suggest that the potential impact of dialysis on brain structure may not linearly increase over time in young patients with CKD. In line with absence of evidence for brain abnormalities in our patients on pre-dialysis therapy, no significant associations were found between eGFR and brain structure. This may indicate that eGFR poorly reflects the concentrations of the wide range of protein-bound uremic toxins that may have a negative impact on the brain [47]. This is the first study to investigate brain structure in young patients on dialysis, presenting evidence suggesting the presence of abnormal white matter integrity and smaller subcortical volume (i.e., nucleus accumbens).

Although positive effects of transplantation on white matter integrity could be anticipated from adult studies [27, 29, 30], findings from our regression analyses suggest that longer time post-transplantation during the patients’ lifetime may be related to more pronounced abnormalities in white matter integrity. To our knowledge, this is the first study to expose this relationship in young CKD patients. Our explorative comparisons of treatment subgroups fit with this finding and suggest that transplanted patients may have widespread disruption of white matter integrity and smaller volume of the nucleus accumbens relative to unaffected peers. These findings align with two recent studies in children and young adults [33, 34]. We found no evidence for striking differences between the pre-emptively transplanted and non-pre-emptively transplanted patients, although this analysis was limited by very small sample sizes. Considering our observations suggesting a role for dialysis therapy in the presence of brain abnormalities, a history of exposure to dialysis may also play a role in the brain abnormalities observed in the transplanted group. However, our findings do suggest that effects of severe CKD on brain structure may not be reversible after transplantation in young patients and may even worsen over time.

The findings of our study suggest that CKD and kidney replacement therapy may impact on axonal integrity. This could imply that white matter abnormalities may become more pronounced over time due to derailed development of white matter tracts after the initial impact of CKD and/or kidney replacement therapy. Moreover, post-transplant infections and neurotoxic/microvascular damage by immune-suppressive maintenance therapy may also contribute to detrimental effects on brain structure after transplantation [11, 13, 48]. The notion that transplanted patients with a normal eGFR, including pre-emptively transplanted patients, and dialysis patients both appeared to have brain abnormalities gives rise to the thought that ischemia induced by impaired cerebral perfusion may play a more important role in the impact of severe CKD on brain structure than uremic toxins. Hypertension, small vessel disease, and impaired cerebral perfusion indeed commonly occur in CKD [5, 14, 49]. This hypothesis fits with the specific structural brain abnormalities revealed in our study, as widespread disruption of white matter integrity has previously been described in adult patients with hypertension, which is in turn associated with impaired cerebral perfusion [50]. This speculation is in line with a recent review, concluding that CKD-related hypertension may be more important as a risk factor for the influence of pediatric CKD on the brain than initially thought [5].

Future studies using a prospective longitudinal design could further clarify the factors that impact on the developing brain, such as duration of CKD, the impact of kidney replacement therapy, course of uncontrolled hypertension, and neurotoxic and vasoactive immuno-suppressive therapy. Obduction or neurophysiological studies may provide further insights in the neuropathology underlying the observed brain abnormalities in patients with CKD. Future research in larger cohorts could take into account the potential roles of co-morbidities and clinical complications of CKD (e.g., premature birth, epilepsy, prolonged severe acidosis). Likewise, longitudinal studies may contribute to better delineation between direct effects of CKD on the brain and secondary effects that may manifest due to derailed brain development. Furthermore, the relevance of structural brain abnormalities for brain function remains to be investigated, for example, in relation to neurocognitive and adaptive functioning in young CKD patients.

### Strengths and limitations

First, we acknowledge our small sample size. Severe CKD is a rare disease in young patients and much effort was done to establish a collaboration with (inter)national child nephrology centers in order to reach as many Dutch-speaking patients as possible. Very cautious interpretation of our findings is necessary, especially with regard to the additional exploratory treatment subgroup analyses. We encourage further investigation of the neuropathology in dialysis and transplanted patients using prospective, long-term longitudinal designs with multiple repeated measurements to increase rigor of the observations and to follow the course of CKD throughout disease and treatment stages. A second limitation is the heterogeneity of our sample in terms of socio-demographic and illness characteristics, which is also partly due to low prevalence of severe CKD in children and young adults. Careful matching of the healthy control group by age, sex, and parental educational level partly accounted for potential group differences and confounding analyses showed that socio-demographic factors did not account for reported group differences. This study also has several strengths, involving the focus on young patients with CKD, the use of a single MRI scanner in an international patient cohort (as multiple scanning sites introduce noise), and the use of advanced quantitative analyses for a comprehensive investigation of brain structure.

## Conclusion and future directions

This study suggests that young patients with severe CKD are at risk of structural brain abnormalities, as detected on MRI by showing widespread abnormality of DTI parameters in the white matter. We further found some evidence suggesting smaller volume of subcortical structures (i.e., nucleus accumbens). Especially patients on dialysis therapy and patients who receive a kidney transplant may be at risk for widespread disruption of white matter integrity and smaller volume of the nucleus accumbens. This study suggests that brain abnormalities in young patients with severe CKD may be partly irreversible, possibly even after successful transplantation. Prospective longitudinal studies should determine the effects of kidney transplantation on the developing brain, which may be less favorable than seen in adult patients.

## Supplementary Information

Below is the link to the electronic supplementary material.

Supplementary file1 (DOCX 25.3 KB)

Supplementary file2 (PPTX 508 KB)

## Abbreviations

AD: Axial diffusivity
ANOVA: Analysis of variance
ATR: Anterior thalamic radiation
CB: Cingulum bundle
CC: Corpus callosum
CKD: Chronic kidney disease
CST: Corticospinal tract
DTI: Diffusion tensor imaging
eGFR: Estimated glomerular filtration rate
FA: Fractional anisotropy
FMa: Forceps major
FMi: Forceps minor
MD: Mean diffusivity
MRI: Magnetic resonance imaging
RD: Radial diffusivity
UF: Uncinate fasciculus
