# Supplementary material for: Structural brain abnormalities in children and young adults with severe chronic kidney disease
Source: Pediatr Nephrol. 2021 Nov 20;37(5):1125–36. doi: 10.1007/s00467-021-05276-5 (PMC9023396; doi:10.1007/s00467-021-05276-5)
Supplement: Supplementary file 1 — Supplementary file1 (DOCX 25.3 KB) [file 467_2021_5276_MOESM1_ESM.docx]

**Supplement 1: specification MRI acquisition and analyses**

#### MRI Acquisition

MRI scans were acquired on a 3.0T Philips Achiva scanner using a 32-channel head coil. T1-weighted images were acquired using a turbo field echo sequence (220 slices, acquisition matrix 240 × 240, voxel-size 1 × 1 × 1 mm, TR/TE/TI = 10/4.20/900 ms, flip angle 8°). Spin echo DWI images were acquired using 128 diffusion gradient directions (b-value = 1000 s/mm^2^, 60 slices, acquisition matrix 112 × 112, TR/TE = 2384.9/75.4 ms, slice thickness = 2 mm) and 1 non-diffusion-weighted volume. Due to a software update during our study, DWI images of two participants were recorded in 32 diffusion gradient directions. Functional MRI of the Brain (FMRIB) Software Library (FSL) version 5.0.11 was used to process all MR images [1].

#### T1-weighted MRI volumetric analyses

Visual inspection of head motion in T1-weigthed images revealed no severe distortion. White matter, gray matter and subcortical structure volumes were estimated using FAST [2] and FIRST [3]. Brain volumes were normalized for head size using the volumetric scaling factor resulting from SIENAX [4]. Volumes of the left and right subcortical structures were combined, since CKD is known to have diffuse impact on the brain [5] and in order to reduce the number of dependent measures and accompanying statistical tests.

#### Diffusion Tensor Imaging (DTI) and Tract Based Spatial Statistics (TBSS)

DTI data of one patient in the pre-dialysis group was excluded due to poor data quality (due to technical difficulties). Pre-processing steps on raw diffusion data included correction for susceptibility induced distortions using *topup*, and artefacts due to subject movement or eddy currents, including automatic detection and imputation of outlier slices (average number of imputed slices 27 [0.3%], range 1–111 [0.0–1.4%) [6, 7]. Removal of non-brain tissue was performed using *Brain Extraction Tool* [8] and masking by non-linear registration of MNI 152 to standard space. Diffusion tensor imaging maps were created using *dtifit* for fractional anisotropy (FA), mean diffusivity (MD), axial diffusivity (AD) and radial diffusivity (RD). Sum of squared error maps were visually inspected to evaluate the quality of the tensor fit.

Voxel-wise statistical analysis of DTI data was performed using tract-based spatial statistics (TBSS) [9]). FA maps for all subjects were warped to MNI152 standard space via the most typical subject in the complete sample of CKD patients and healthy control group. The analyses considering the association with CKD parameters could not be performed in the complete sample and therefore the selection of the most typical subject was done in CKD patients only. A white matter skeleton was computed to confine the analysis to the regions of white matter tracts with minimal neuroanatomical variability (FA > 0.3). At last, MD, AD and RD maps were also projected on this skeleton.

Voxel-wise statistical comparisons were performed using the *randomize* tool in FSL on DTI maps, where threshold-free cluster enhancement and family-wise error corrected *p*-values accounted for multiple testing. The FSL John Hopkins University atlases were used to identify the white matter regions involved in significant voxel clusters [10].

**References**

1. Jenkinson M, Beckmann CF, Behrens TEJ, Woolrich MW, Smith SM (2012) FSL. Neuroimage 62:782-790. https://doi.org/10.1016/j.neuroimage.2011.09.015

2. Zhang Y, Brady M, Smith S (2001) Segmentation of brain MR images through a hidden Markov random field model and the expectation-maximization algorithm. IEEE Trans Med Imaging 20:45-57 https://doi.org/10.1109/42.906424

3. Patenaude B, Smith SM, Kennedy DN, Jenkinson M (2011) A Bayesian model of shape and appearance for subcortical brain segmentation. Neuroimage 56:907-922. https://doi.org/10.1016/j.neuroimage.2011.02.046

4. Smith SM, Zhang Y, Jenkinson M, Chen J, Matthews PM, Federico A, De Stefano N (2002) Accurate, robust, and automated longitudinal and cross-sectional brain change analysis. Neuroimage 17:479-489

5. Jabbari B, Vaziri ND (2018) The nature, consequences, and management of neurological disorders in chronic kidney disease. Hemodial Int 22:150-160. https://doi.org/10.1111/hdi.12587

6. Andersson JL, Skare S, Ashburner J (2003) How to correct susceptibility distortions in spin-echo echo-planar images: application to diffusion tensor imaging. Neuroimage 20:870-888. https://doi.org/10.1016/s1053-8119(03)00336-7

7. Andersson JLR, Sotiropoulos SN (2016) An integrated approach to correction for off-resonance effects and subject movement in diffusion MR imaging. Neuroimage 125:1063-1078. https://doi.org/10.1016/j.neuroimage.2015.10.019

8. Smith SM (2002) Fast robust automated brain extraction. Hum Brain Mapp 17:143-155. https://doi.org/10.1002/hbm.10062

9. Smith SM, Jenkinson M, Johansen-Berg H, Rueckert D et al (2006) Tract-based spatial statistics: voxelwise analysis of multi-subject diffusion data. Neuroimage 31:1487-1505. https://doi.org/10.1016/j.neuroimage.2006.02.024

10. Mori S, Oishi K, Jiang H, Jiang L et al (2008) Stereotaxic white matter atlas based on diffusion tensor imaging in an ICBM template. Neuroimage 40:570-582. https://doi.org/10.1016/j.neuroimage.2007.12.035
