# Supplementary material for: Structural brain abnormalities in children and young adults with severe chronic kidney disease
Source: Pediatr Nephrol. 2021 Nov 20;37(5):1125–36. doi: 10.1007/s00467-021-05276-5 (PMC9023396; doi:10.1007/s00467-021-05276-5)
Supplement: Supplementary file 2 — Supplementary file2 (PPTX 508 KB) [file 467_2021_5276_MOESM2_ESM.pptx]

## Slide 1
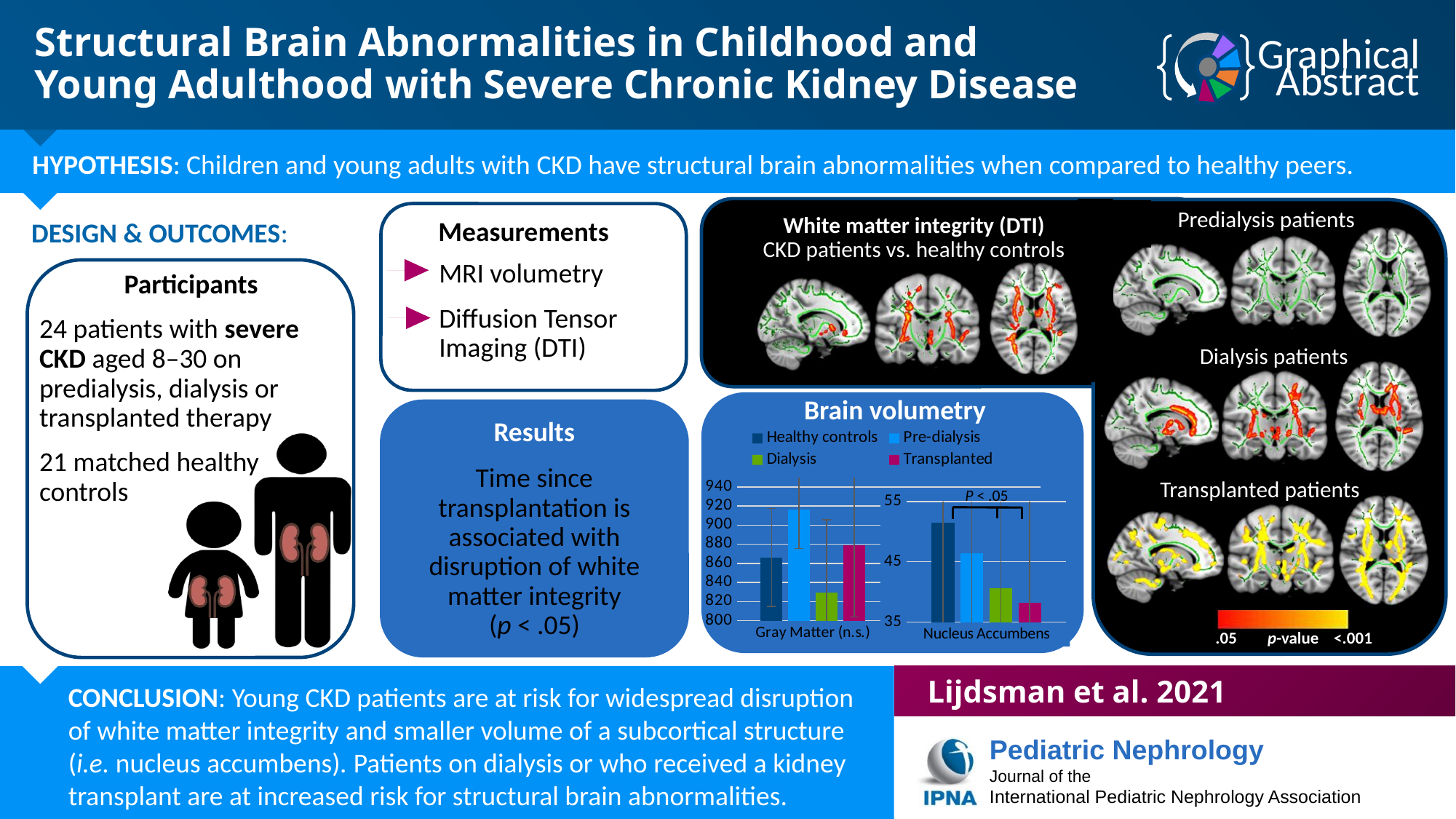

Structural Brain Abnormalities in Childhood and Young Adulthood with Severe Chronic Kidney Disease
HYPOTHESIS: Children and young adults with CKD have structural brain abnormalities when compared to healthy peers.
White matter integrity (DTI)
CKD patients vs. healthy controls
Predialysis patients
Dialysis patients
Transplanted patients
.05 p-value <.001
Measurements
DESIGN & OUTCOMES:
MRI volumetry
Diffusion Tensor Imaging (DTI)
Participants
24 patients with severe CKD aged 8–30 on predialysis, dialysis or transplanted therapy
21 matched healthy controls
Brain volumetry
### Chart
| Category | Healthy controls | Pre-dialysis | Dialysis | Transplanted |
|---|---|---|---|---|
| Gray Matter (n.s.) | 866.0 | 916.0 | 829.0 | 879.0 |
### Chart
| Category | Healthy controls | Pre-dialysis | Dialysis | Transplanted |
|---|---|---|---|---|
| Nucleus Accumbens | 51.5 | 46.4 | 40.6 | 38.2 |
Results
Time since transplantation is associated with disruption of white matter integrity
(p < .05)
P < .05
Lijdsman et al. 2021
CONCLUSION: Young CKD patients are at risk for widespread disruption of white matter integrity and smaller volume of a subcortical structure (i.e. nucleus accumbens). Patients on dialysis or who received a kidney transplant are at increased risk for structural brain abnormalities.
